# Panelist

**DOI:** 10.1002/alz70856_106087

**Published:** 2026-01-08

**Authors:** Ronald Petersen

**Affiliations:** ^1^ Mayo Clinic, Rochester, MN, USA

## Abstract

Dr. Petersen will be a panelist.

